# Parallel Placement of Excluder Legs to Treat Abdominal Aortic Aneurysms with Aortoiliac Occlusive Lesion

**DOI:** 10.3400/avd.cr.20-00044

**Published:** 2020-09-25

**Authors:** Hiroaki Kato, Noriyuki Kato, Ken Nakajima, Takatoshi Higashigawa, Takafumi Ouchi, Shuji Chino, Toshiya Tokui, Hajime Sakuma

**Affiliations:** 1 Department of Radiology, Mie University Hospital; 2 Department of Radiology, Ise Red Cross Hospital; 3 Department of Cardiovascular Surgery, Ise Red Cross Hospital

**Keywords:** endovascular aneurysm repair (EVAR), parallel placement, abdominal aorta

## Abstract

The effectiveness of endovascular aneurysm repair (EVAR) has been proven, but anatomical limitations, including narrow access route, may obstruct procedure of EVAR and cause serious complications. Parallel placement of Excluder legs (W. L. Gore & Associates, Inc., Newark, DE, USA) was established to treat patients with type IIIb endoleak or those with a narrow aorta, who could not be treated using a standard main body. In this report, we applied this technique in two patients with aortoiliac aneurysms with occlusive lesion.

## Introduction

Although endovascular aneurysm repair (EVAR) has been established as a mainstream approach to treat aortoiliac aneurysm, there are still some anatomical limitations.^1,2)^ One such anatomical limitation is a narrow access route, which can make EVAR unfeasible using a standard main body. In this report, our experience with the parallel placement of Gore Excluder legs in two patients with aortoiliac aneurysms with a narrow access route has been described. Our institutional review board (No. 1717) approved this case report.

## Case Report

### Case 1

**Figure 1:**
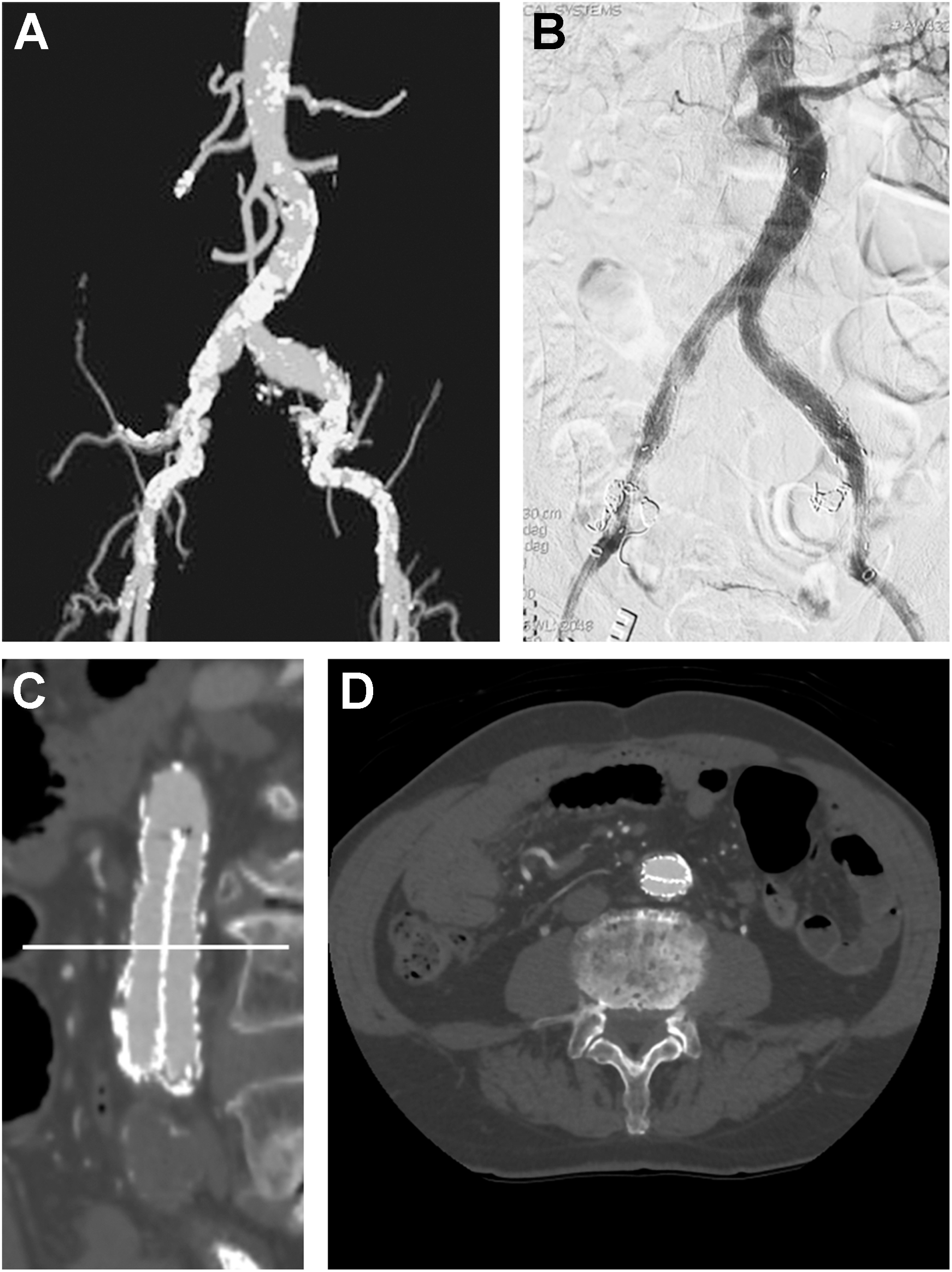
Fig. 1 (**A**) Maximum intensity projection image of preoperative contrast-enhanced computed tomography (CT) shows marked calcification of both external iliac arteries. (**B**) Completion angiogram shows no endoleak after parallel placement of Excluder legs. (**C**) The multi-planar reconstruction image of postoperative CT shows parallel configuration of Gore Excluder legs inside the abdominal aorta. The white horizontal line shows the level of the axial image (**D**). (**D**) The axial image shows complete sealing and no collapse of legs.

A 78-year-old man with bilateral common iliac arterial aneurysms was referred to us. The diameter of the aneurysm was 44 mm on the left side and 21 mm on the right side. Coil embolization of the left internal iliac artery (IIA) was conducted first. One month later, coil embolization of the right IIA was conducted, and EVAR using an Excluder main body (W. L. Gore & Associates, Inc., Newark, DE, USA) was attempted. However, EVAR using a main body was abandoned. Instead, the parallel placement of Excluder legs was attempted because a 16 Fr DrySeal sheath (W. L. Gore & Associates, Inc.) could not be advanced beyond the heavily calcified external iliac artery (EIA) (**Fig. 1A**). After dilation of the stenotic site using a balloon catheter, two 12 Fr DrySeal sheaths could be advanced. The diameter of the Excluder legs used for parallel placement was calculated by dividing half the circumference of the proximal neck plus its diameter by the circular constant. To minimize the potential gutter endoleaks arising between the two legs, an oversized diameter of >10% of the diameter mentioned above, which was 16 mm, was judged as appropriate to obtain sealing at the aorta. Then, the Excluder legs with a 12 mm bottom diameter and a 14 cm length were inserted into each sheath. They were then placed from just below the renal orifice down to both the common iliac arteries (CIAs). Iliac extenders were added to extend the covered segment to EIAs. Completion angiogram showed complete exclusion of the aneurysms (**Fig. 1B**), and cone beam computed tomography (CT) showed no collapse on either leg (**Figs. 1C** and **1D**). Follow-up CT obtained 2 years later showed no endoleak and shrinkage of the aneurysms from 44 to 40 mm on the left side and from 21 to 18 mm on the right side.

### Case 2

**Figure 2:**
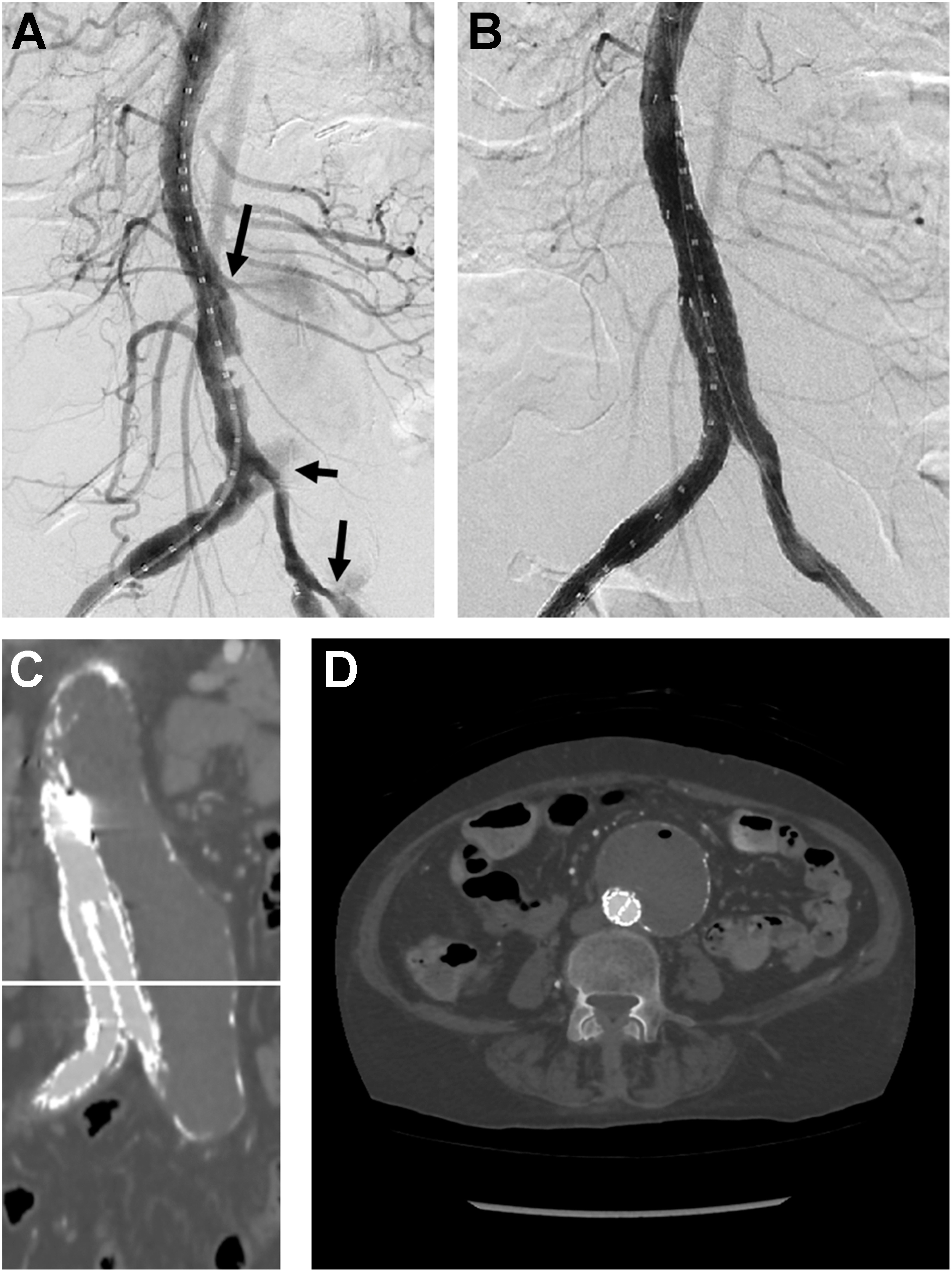
Fig. 2 (**A**) The initial angiogram shows three communications between the true and false lumen (arrows). (**B**) The completion angiogram shows no endoleak. (**C**, **D**) Multi-planar reconstruction image of postoperative computed tomography shows parallel configuration of Gore Excluder legs. The white horizontal line shows the level of the axial image (**D**). (**D**) The axial image shows no endoleak or collapse of legs.

A 64-year-old woman diagnosed with an impending rupture of the thoracoabdominal aortic aneurysms measuring 59 mm in the descending thoracic aorta and 56 mm in the abdominal aorta (**Fig. 2A**) was transferred to our hospital. She had history of ascending aorta and aortic arch replacement using a frozen elephant trunk for the treatment of type A aortic dissection. She experienced renal failure and was on regular hemodialysis. Emergent contrast-enhanced CT revealed fenestrations between the true and false lumen in the lower abdominal aorta, right CIA, and left EIA. Closure of these fenestrations using stent grafts was proposed. EVAR using a standard main body was considered difficult because the diameter of the true lumen in the abdominal aorta was 13 mm, and that of both the EIAs was only 4 mm; parallel placement of Excluder legs was selected. After embolization of the left IIA using Amplatz Vascular Plug IV (St. Jude Medical, St. Paul, MN, USA), an Excluder leg (bottom diameter, 16 mm; length, 10 cm) was placed through a 14 Fr DrySeal sheath with the distal end just above the aortic bifurcation to close the fenestration in the lower abdominal aorta. Two 14 Fr DrySeal sheaths were then advanced from both the femoral arteries into the Excluder leg placed beforehand. Two Excluder legs (bottom diameter, 12 mm; length, 14 cm) were deployed at the same level, proximally overlapping the previously placed Excluder leg by 4 cm. An iliac extender was added to completely close the fenestration in the left EIA. Because type Ia endoleak was observed, coil embolization of the tract and placement of a 23×30 Excluder cuff on the proximal end were added. Completion angiogram showed no endoleak (**Fig. 2B**). The 18 month follow-up period was uneventful (**Figs. 2C** and **2D**), and the diameter of the descending thoracic aorta and abdominal aorta decreased from 59 to 55 mm and 56 to 53 mm, respectively.

## Discussion

In most of the presently available devices for EVAR, the delivery system profile ranges from 16 Fr to 22 Fr; therefore, the access route diameter must be at least >5 mm. Even if smaller than 5 mm, the elastic artery would allow the introduction of the delivery system. However, access route injury may develop in a heavily calcified artery even if the diameter of the access route is larger than that of the delivery system. In 0.9% of patients in the EUROSTAR registry, conversion to open repair was required because of access-related complications.^3)^ In the DREAM trial, the frequency of local vascular or implant-related complications was >16%, and that of severe complications was approximately 4%.^1)^ Several options are available to avoid these complications. One such option is to use the latest devices with smaller profiles, such as Ovation (Endologix, Inc., Irvine, CA, USA). The outer diameter of this device (14 Fr or 16 Fr) would allow most patients to be treated without any access route complications. However, the long and stiff suprarenal stent of this device should select patients, especially in a case with a tortuous suprarenal aorta. Other options include the use of open iliac conduits or internal endoconduits, both of which may be accompanied by potentially serious complications.^4)^ By contrast, the parallel placement of Excluder legs requires only two DrySeal sheaths with profiles ranging from 12 Fr to 16 Fr. Similar to our patients, the eligibility of EVAR in patients with a narrow access route would be increased by the availability of these low-profile sheaths.

A rare but serious postoperative complication is limb occlusion. It has been reported that it develops in<4% of patients who are undergoing EVAR.^5)^ There are several causes of limb occlusion^6)^; one of these is being in a narrow aorta. When the contralateral limb is located in the narrow aorta, the ipsilateral limb of a standard main body could be compressed by the other limb because of overlapping, which can cause limb occlusion. An aortouniiliac stent graft could be a solution in such a case; however, it necessitates the embolization of the contralateral iliac artery and femorofemoral bypass. Lepidi et al. obtained excellent results by applying the parallel placement of Excluder legs in 18 patients with distal aortic and common iliac aneurysms.^7)^ In our report, parallel placement of Excluder legs was used in Case 2 because of both narrow access routes and a narrow aorta, which made the use of a standard main body difficult.

Parallel placement of Excluder legs was first reported by Reijnen et al. to treat a type IIIb endoleak.^8)^ Since then, the procedure has been adopted by several researchers because it does not require the use of any special devices.^7,9)^ However, this technique has a few potential drawbacks; one of these is the development of gutter endoleaks. Even if the tops of the two Excluder legs are placed at the same level, the rest of the legs are not always parallel to each other, which can create a gutter between them. Another drawback is that compared with the standard main body, the parallel placement lacks device-fixing mechanisms, which can cause device migration. Thus, close follow-up is a must.

## Conclusion

To conclude, parallel placement appears to be a promising technique for EVAR in patients with a narrow access route; however, before it can be widely used, further investigations are needed.
